# A school-based interdisciplinary approach to promote health and academic achievement among children in a deprived neighborhood: study protocol for a mixed-methods evaluation

**DOI:** 10.1186/s12889-018-5309-9

**Published:** 2018-04-10

**Authors:** Mariëlle E. Abrahamse, Caroline S. Jonkman, Janneke Harting

**Affiliations:** 1 Department of Public Health, Academic Medical Center, University of Amsterdam, Amsterdam, The Netherlands; 2 Department of Clinical Child and Family Studies and the EMGO Institute for Health and Care Research, VU University, Amsterdam, The Netherlands

**Keywords:** Health, Poverty, Academic achievement, Children, Primary school intervention, Interdisciplinary approach

## Abstract

**Background:**

The large number of children that grow up in poverty is concerning, especially given the negative developmental outcomes that can persist into adulthood. Poverty has been found as a risk factor to negatively affect academic achievement and health outcomes in children. Interdisciplinary interventions can be an effective way to promote health and academic achievement. The present study aims to evaluate a school-based interdisciplinary approach on child health, poverty, and academic achievement using a mixed-method design. Generally taken, outcomes of this study increase the knowledge about effective ways to give disadvantaged children equal chances early in their lives.

**Methods:**

An observational study with a mixed-methods design including both quantitative and qualitative data collection methods will be used to evaluate the interdisciplinary approach. The overall research project exists of three study parts including a longitudinal study, a cross-sectional study, and a process evaluation. Using a multi-source approach we will assess child health as the primary outcome. Child poverty and child academic achievement will be assessed as secondary outcomes. The process evaluation will observe the program’s effects on the school environment and the program’s implementation in order to obtain more knowledge on how to disseminate the interdisciplinary approach to other schools and neighborhoods.

**Discussion:**

The implementation of a school-based interdisciplinary approach via primary schools combining the cross-sectoral domains health, poverty, and academic achievement is innovative and a step forward to reach an ethnic minority population. However, the large variety of the interventions and activities within the approach can limit the validity of the study. Including a process evaluation will therefore help to improve the interpretation of our findings. In order to contribute to policy and practice focusing on decreasing the unequal chances of children growing up in deprived neighborhoods, it is important to study whether the intervention leads to positive developmental outcomes in children.

**Trial registration:**

(NTR 6571) (retrospectively registered on August 4, 2017).

## Background

Children growing up in neighborhoods with high levels of poverty are at risk for negative developmental outcomes and inequality of their chances in life e.g., [1, 2]. Although children in the Netherlands have on average the lowest risk (11.9%) on poverty in the European Union, the number of children at risk of poverty on the three largest Dutch cities is still relatively high [3]. In Amsterdam, 1 on 4 children is at risk for growing up in poverty. On neighborhood level this number is even higher with regard to deprived neighborhoods in Amsterdam. Therefore, a substantial number of children in the Amsterdam neighborhood Holendrecht grow up in poverty. In this neighborhood already 27% of the households have an income up to 120% of the social minimum [4].

During the past decades, a large body of research has indicated the negative effects of childhood poverty on child health, well-being, and other developmental outcomes such as academic achievement. Yoshikawa, Aber, and Beardslee [5] outlined a conceptual framework emphasizing how poverty can deeply impact the emotional and behavioral health of children through a variety of mediating mechanisms. For example, children who grow up in low-income families are four times more likely to be in fair or poor health. Also, child obesity is more prevalent among children from low-income families compared to their more affluent peers [6]. With regard to mental health, children growing up in low-income households tend to express more emotional and conduct problems which is linked to impaired family functioning as a consequence of economic stress [7]. Recently, it was even shown that childhood poverty impacts the child’s brain development [8]. Another important factor rising the inequality of children’s chances in life is that the schools in disadvantaged neighborhoods tend to suffer from a variety of factors negatively affecting their quality and leading to lower academic achievement [9, 10]. For instance, schools with a large proportion of students from low-income families have more often less-effective teachers and a greater number of in-class disruptions than schools with a lower number of low-income students [10, 11].

The negative effects of growing up in poverty during childhood tend to persist into adulthood. Adults who were poor during childhood are more likely to by poor during adolescence and adulthood than those who were never poor. Also, studies have shown that children living in poverty early in their lives are at risk for a variety of negative outcomes in adulthood that impact the society, such as more criminal activities, poorer overall health, and more psychological distress [12]. Because of these long-term consequences for children growing up in poverty, early intervention is of outmost importance.

To promote equal chances in life for all children regardless of their socio-economic background, Gemeente Amsterdam (municipality of Amsterdam) initiated a school-based interdisciplinary intervention, named Stadsscholen020. In this intervention approach different policy area’s collaborate together, such as the departments focusing on poverty reduction, youth, and education. This can be seen as an integrated school (health) policy. Increasing the educational quality at schools is necessary to improve learning in children and reduce the consequences of childhood poverty, but at the same time research has shown that it is insufficient to create equal chances for children growing up in poverty compared to their more affluent peers [13]. Therefore, an interdisciplinary school-based approach that addresses behavior in a broader social context is thought to be more effective to prevent the negative outcomes related to poverty and growing up in disadvantaged neighborhoods [14]. Beside the focus on educational quality, the current intervention uses an cross-sectoral approach with poverty and health. Including other related domains embedded in the multi-component intervention is leading to an integrated school health policy. Another benefit of this school-based program is that the universal enrollment of children in these programs tend to have the potential to reach a larger group of children. This can lead to behavioral changes in a group of low-income children that is often hard to reach for individual intervention. Also, these behavioral changes can positively affect their development into adolescence and adulthood [15–17].

Although interdisciplinary school-based programs are widely used and researched, most programs focus on promoting healthy lifestyles, physical activity and obesity prevention among children [18]. The current intervention approach is slightly different because of the primary focus on equal educational chances by a collaboration with different policy areas. However, literature on school-based interdisciplinary programs focusing on broader outcomes and using similar activities is limited. Because a successful implementation of this intervention is complex and requires a lot of effort of the professionals involved, it is yet unknown how effective this intervention strategy is.

The purpose of the current research project is to study the effects and evaluate the process of Stadsscholen020 on child poverty, academic achievement, and health-related outcomes using a observational research design. We will use a convergent mixed-methods design, where quantitative and qualitative research are conducted and analyzed both separately and combined for interpretation purposes [19, 20]. As suggested by recent guidelines for the evaluation of complex interventions our study also includes a process evaluation as suggested in recent guidelines for the evaluation of complex interventions [21]. Generally taken, with our findings we tend to increase the knowledge about effective ways to give disadvantaged children equal chances from an early age.

## Methods

### Design and study setting

The current study is an observational study within four primary schools located in Holendrecht, Amsterdam. Holendrecht is a neighborhood in the south eastern part of Amsterdam. Since the intervention is innovative and is being developed during the intervention period, we have chosen for this mixed-methods observational design. Stadsscholen020 is a new and complex intervention approach that comprises multiple interacting components and therefore we aim to evaluate policy, practice, and the implementation directly from the start of the intervention. Based on the mixed-methods design, our current study can be divided into three slightly overlapping and complementary study parts. First, we will conduct a longitudinal study (Part I) to evaluate the effects on the individual level for children using five assessment points. Second, in a cross-sectional study (Part II) we will evaluate the effects on school and neighborhood level. Third, using a process evaluation (Part III) we will study the implementation of the intervention and interpret our findings of the other study parts. For each study part different methods and instruments are used with regards to the data collection.

### Eligibility criteria

In the initial pilot year (2015–2016) only four primary schools in Amsterdam Holendrecht neighborhood were focus of Stadsscholen020. Currently, eight other schools from deprived neighborhoods in Amsterdam are involved. The so called ‘Stadsscholen’ are selected based on their increased risk for educational disadvantages due to challenges and difficulties on family and neighborhood level (based on unpublished data from the municipality). Although there are more schools involved in the Stadsscholen020 program, for this research study we have chosen to focus on the four schools in the neighborhood Holendrecht since the interdisciplinary approach initially started at these schools. Because interdisciplinary approaches are time-consuming to implement in a school setting and it takes time to observe the potential effects, we have chosen for the schools with the longest intervention period. Also, our decision to focus on the four schools is based on considerations to limit the complexity of our study and conduct an in-depth process evaluation within the time frame of our study.

### Participants and recruitment

For the longitudinal part and process evaluation of our study we will recruit participants. The cross-sectional part of the study only includes generally collected data whereby recruitment of participants is not applicable. For the longitudinal part of our study, children in the so called groups 5 to 8 are enrolled in the study, which is internationally comparable with children in the 3rd to 6th grade. In these ‘groups’ children have the age to be able to validly complete self-report questionnaires. In the Dutch educational system, children attend primary school from the age of 4 until the age of 11 or 12, where they proceed to secondary school.

During the intervention period, the children of the participating schools (approximately *N* = 475) are asked to complete the questionnaires during five assessment points. Therefore, the population will consist of a dynamic cohort throughout the school years (Table 1). Parents are informed about the study with a school letter and an announcement in the school newsletter. Also, the researcher will be present at the school or during parent meetings to inform them about the study. In case parents don’t want their child to participate in the research study, they can inform the school or the researcher (opting-out procedure). The child’s questionnaires will be administered during the regular classes where a member of the research team is available to ensure independent and confidential responding and to provide assistance when necessary.

**Table 1:** Overview longitudinal cohort assessments

Cohort	T1 (Sep-Nov 2016)	T2 (May-Jun 2017)	T3 (Sep-Nov 2016)	T4 (May-Jun 2018)	T5 (Sep-Nov 2018)
A					Group 5
B			Group 5	Group 5	Group 6
C	Group 5	Group 5	Group 6	Group 6	Group 7
D	Group 6	Group 6	Group 7	Group 7	Group 8
E	Group 7	Group 7	Group 8	Group 8	
F	Group 8	Group 8			

The population of the process evaluation includes participants on school-level and family-level. All educational staff working at the four primary schools are invited to participate anonymously by completing a questionnaire five times during the study period. These time points are similar to the assessments of the children and will be administered on paper or using an online survey platform. Also, the schoolboard, parents, and policy and practice stakeholders of the municipality of Amsterdam will be included as a participant in this process evaluation.

### Intervention

The initial purpose of the Stadsscholen020 intervention program initiated by the municipality of Amsterdam is to promote equal chances for a successful educational career for all children who grow up in Amsterdam regardless their ethnic background, family situation, and neighborhood. The intervention period of Stadsscholen020 is two school years starting in September 2016 until July 2018. The intervention started right after the pilot phase during school year 2015–2016 which can be seen as an orientation period for introducing and developing the activities of Stadsscholen020. The primary schools participating in this school-based interdisciplinary intervention approach received a financial impulse to have extra resources for the three following domains: Supporting the school team, Improving student learning, and Supporting and creating more resources around the school.

#### School specific activities

The schoolboards are in the lead to spend the extra financial resources on staff, programs, or activities based on their own view what is needed to reach the initial purpose of Stadsscholen020. Therefore, the four schools use the financial impulse differently. They describe and justify their decisions and vision in a customized developmental plan (Maatwerkplan). Although there is a large variety among the choices for activities and interventions at the four schools in Holendrecht, most of them have a focus on similar themes and goals that are linked to the general three domains. Most frequently there is a focus on 1) increasing educational quality using teacher mentors (domain; Supporting the school team), 2) supporting the school team by hiring additional educational and management staff (Supporting the school team), 3) increasing parental involvement for their child’s education (Supporting and creating more resources around the school), and 4) improving personal and skill development for students (Improving student learning).

#### Overarching general activities

Beside the school’s specific activities, the Stadsscholen020 program contains some overarching activities in which all four schools participate. For example, Stadsscholen020 started a pilot where parents can pay the parental school fee with a permit available to low-income families (Stadspas). Often, low-income parents cannot afford payment of the parental school free, which is used for school activities and trips. To prevent children from exclusion of social and learning activities, the pilot – wherein the parental fee is paid with the permit–, schools are compensated by municipality finances. Another example is a Community Project initiated by an international business company (AkzoNobel) to help children getting more familiar with business life and have young successful people as a role model for their future careers. Also, Stadsscholen020 started organizing a Graduation Party for children in Group 8 (6th grade) at the end of the educational year to highlight the children’s accomplishments during their primary school trajectory, to support them with their next step to secondary education, and to increase parental involvement in the child’s academic achievements.

#### Health promotion component

Beside the financial impulse of the municipality of Amsterdam, an additional grant is available for each of the four schools to focus specifically on health promotion for the children. This grant was made available by the Fonds NutsOhra, a funding organization supporting initiatives that promote the quality of life of vulnerable groups in the Dutch society. Each school receives additional financial resources to spend on interventions for emotional and/or physical health promotion that will be integrated within the Stadsscholen020 program. Again, the schoolboards are in the lead to choose an intervention that fits the school’s vision on health. For example, the schools are able to invest in school-based positive behavior interventions or healthy school-based food and nutrition policies.

### Outcomes

The primary outcome in the current study is child health. This outcome will be assessed using a mixed-methods approach to measure child health indicators. Also, a variety of indicators will be used to assess the secondary outcomes. The secondary outcomes are child poverty and child academic achievement. Both qualitative and quantitative data on outcome measures will be collected in the overlapping study parts. Table 2 presents an overview of all quantitative data collected for the outcome measures including the informants and measurements.

#### Part I. Longitudinal study

Because of the interdisciplinary character of the Stadsscholen020 program, the variety of interventions and activities may bring positive changes on indicators related to child health, poverty, and academic achievement. Therefore, we have chosen to measure a number of indicators related to these outcomes using self-report questionnaires for children. Measures include standardized questionnaires that were previously used in similar populations. A number of supplementary questions are added to these standardized questionnaires in order to collect additional data related to the indicators of the outcomes. For example, with regards to physical health, children are asked about sport club membership and dietary behavior.

##### I.1 Child Health

*Life satisfaction.* Life satisfaction is an important component of the child’s overall wellbeing since childhood life satisfaction is related to a number positive outcomes in adolescence and adulthood [22]. Children are asked to grade their lives on the 11-step Cantril Ladder where 0 is indicating the worst possible life and 10 the best possible life [23].

*Psychosocial health.* The Strengths and Difficulties Questionnaire (SDQ) is a brief questionnaire that measures the psychosocial adjustment of children and adolescents [24]. In the current study we use the 25-items self-report version for children aged 11–16 years old. Since not all children in our sample already reached the age of 11, only children in Group 7 and Group 8 will complete this questionnaire. The SDQ consists of five dimensions of difficulties, Emotional Problems, Conduct Problems, Hyperactivity, Peer Relations, Total Difficulties, and one dimension of strengths (Prosocial Behavior) rated on a three-point scale (0 = *not true*, 1 = *somewhat true,* 2 = *very true*). The reliability and validity of the SDQ self-report have been found satisfactory in a Dutch primary school sample [25].

*Quality of life.* The KIDSCREEN measures the health-related quality of life in children and adolescents (8 to 18 years). Children fill out the shortened version of the original KIDSCREEN – 52, including the following dimensions: Physical Well-being, Psychological Well-being, Moods & Emotions, Self-perception, Autonomy, Parent Relations, Social Support and Peers, School Environment, and Social Acceptance (Bullying). To identify the global unidimensional health status of children, the KIDSCREEN – 10 index scale is used. All items are rated at a five-point scale (1 = *not at all*, 2 = *slightly*, 3 = *moderately*, 4 = *very*, 5 = *extremely*). The KIDSCREEN has good psychometric properties among primary school aged children (from the age of 8) and will therefore be administrated with children in Group 5 to 8 [26].

##### I.2  Child Poverty

*Socioeconomic position.* To measure the socioeconomic status of the children at the schools, an adapted version of the Family Affluence Scale (FAS III) will be used [27, 28]. Children are asked about socioeconomic indicators that they are likely to know about their family and home situation. The scale includes questions about car ownership, if they have an unshared bedroom for their own, and if they traveled last year with their family for a holiday abroad. Based on recent suggestions for a revised scale [28], children are also asked about the presence of a washing machine, tumble dryer, and dishwasher in their homes. The full composite scale will be used as an continuous variable in order to test for differences between assessment points. Also, we intent to distinguish low, middle, and high affluence groups using tertiles. Additionally, we also added some questions that were previously used in Dutch research focusing on childhood poverty. Children are asked if they celebrate their birthday every year and if they eat fruit and have warm dinner every day [29]. Also, children are asked if they own a Stadpas, an activity and cultural permit funded by the municipality of Amsterdam that children receive if the family income is below 120% of the social minimum. This question would be an indicator for low-income households.

#### Part II. Cross-sectional study

Complementary to the individual data and detailed information from the longitudinal and process evaluation, the cross-sectional study will include data from public databases of the municipality of Amsterdam (Onderzoek, Informatie en Statistiek, OIS), the Public Health Service of Amsterdam (GGD), and The Education Executive Agency of the Dutch Ministry of Education, Culture, and Science (Dienst Uitvoering Onderwijs, DUO). These data will be used to observe the effects of Stadsscholen020 during the intervention period on determinants related to health, poverty, and academic achievement that are available on school and neighborhood level. Data from the schools and the neighborhood where the schools are located (Amsterdam Holendrecht) will be compared with data on a wider district and city level. Table 2 shows the overview of the indicators related to the primary and secondary outcomes that will be examined in this cross-sectional study.

**Table 2:** Overview of primary and secondary outcomes, indicators, and measurements

	Informants	Study parts		
		Longitudinal study *5 assessments*	Cross-sectional study *5 year period*	Process evaluation *5 assessments*
*Child Health*				
Life satisfaction (Cantril Ladder)	Child	×	×	
Socioemotional Well-being (SDQ)	Child	×		
Quality of life (KIDSCREEN)	Child	×		
Sport club membership	Child	×		
Dietary behavior (daily warm meal and fruit intake questions)	Child	×		
% 5-years old / 10-years old children with overweight (including obesity)	GGD		×	
% 5-years old / 10-years old children having breakfast every day	GGD		×	
% 5-years old / 10-years old children eating greens every day	GGD		×	
% 5-years old / 10-years old children that is physical active	GGD		×	
% Households supported by child welfare organizations (OKT)	GGD		×	
*Child Poverty*				
Family Affluence (FAS)	Child	×		
Stadspas ownership (minimal income permit)	Child	×		
% Households depending on social security benefits	OIS		×	
% Households having an income up to 120% of the social minimum	OIS		×	
% Children having parents with a lower education	OIS		×	
*Child Academic Achievement*				
Secondary school qualifications	DUO		×	
School test results (CITO)	DUO		×	
*School Environment*				
Teacher self-efficacy (TSES)	Teacher			×
Classroom climate (Class climate scale)	Teacher			×
School and neighborhood safety (additional questions)	Child			×
Bullying (KIDSCREEN and additional questions)	Child			×
School subscale (KIDSCREEN)	Child			×

Abbreviations: *SDQ* Strengths and Difficulties Questionnaire, *GGD* Public Health Service of Amsterdam, *OKT* ouder-kind team (parent-child team), *FAS* Family Affluence Scale, *CITO* Centrale Eindtoets Basisonderwijs (Final Examination for Primary Education), *OIS* Department of Research, Information, and Statistics of the municipality of Amsterdam, *DUO* Dienst Uitvoering Onderwijs (The Education Executive Agency), *TSES* Teacher Sense of Efficacy Scale

#### Part III. Process evaluation

Since Stadsscholen020 is a complex interdisciplinary approach with a large variety of activities and interventions, a process evaluation will help to provide more detailed information about the effectiveness of the program. The process evaluation will be conducted to provide insight on the reach, acceptance, implementation, and feasibility of the intervention at the schools. Also, the process evaluation will give guidance for future successful implementation of intervention components at other schools and neighbourhoods in the Netherlands. Thereby, the process evaluation will help to understand the findings from the longitudinal and cross-sectional study parts.

Part of the process evaluation will include a detailed description of all the interventions and activities at the four schools initiated with the supplemental financial resources. With regards to the effectiveness of Stadsscholen020 this description provides information about the mechanisms that impact the outcomes. The process evaluation will include both quantitative and qualitative methods. Qualitative interviews with the policy and practice stakeholders, members of the school team, and parents. For example, the school boards will be interviewed several times during the intervention period to reflect on the implementation process of the program. Also, the potential benefits for the school, teachers, and children are discussed in this interview. Additionally, project members and policy makers of the municipality of Amsterdam will be interviewed about their experiences with Staddscholen020. These qualitative interviews will be recorded, transcribed, and subsequently coded using qualitative data analysis software (MAXQDA). In addition to the qualitative interviews, the documentary collected during the intervention period will be analysed. These documentary includes policy documents, minutes of meetings, and self-administered logs of observations conducted by the research team.

Quantitative data for the process evaluation will be collected from children and teachers with regards to the school environment as a mechanism that impact the outcomes on child health and academic achievement. In joined collaboration with involved parents at the schools, supplementary questions on the school- and neighbourhood safety are included in the child questionnaire. Also, two subscales of the KIDSCREEN (School Environment and Bullying) are used to obtain information about the school environment. With regards to the teacher’s perception of the school environment, the following two questionnaires are additionally administered:

##### III.1 School Environment

*Teacher Self-efficacy.* School performance of children can be positively affected by the teacher’s self-efficacy [30]. Therefore, we will use teacher self-efficacy as in indicator of child academic achievement. The Dutch translation of the Teacher Sense of Efficacy Scale (TSES) is used [31, 32]. The TSES measures the teachers’ perceptions of their competence across a range of teaching tasks. For the current study we use the shortened version that consists of 12 items. Items are rated at a 7-point scale, ranging from 1 (*nothing*) to 7 (*a great deal*). Psychometric properties of the TSES have been found adequate [32].

*Classroom climate.* The ‘Klimaatschaal’ (Class climate scale) is a Dutch questionnaire to measure the classroom’s pedagogical environment assessed by teachers [33]. There are four dimensions that constitute the pedagogical environment: Quality of Mutual Student Relationships, Class Atmosphere, Enforcement, and Quality of the Student-teacher Interaction. The questionnaire consists of 33 items rated at a 4-point scale (1 = *[almost] never*, 2 = *sometimes*, 3 = *regularly*, 4 = *always*). The ‘Klimaatschaal’ has adequate psychometric properties [33].

### Statistical analyses

#### Power calculation for the primary outcome

At the start of our study the four schools have respectively 82, 119, 121, and 155 children in the targeted groups (Group 5 to 8; 477 children in total for the four schools). Because each school has different activities and interventions related to the Stadsscholen020 program we conducted a power calculation to estimate the sample size per school to ensure there is enough power to detect intervention effects over time. For this power calculation we used the general health-related quality of life in children (KIDSCREEN-10 index) as an indicator of our primary outcome Child Health. Using norm data [26] and pilot data from our sample we calculated that an effect size of 0.70 is related to a relevant change. Based on Cohen’s *d* this effect size is viewed as a large effect [34]. A sample size of 56 children per school was required to detect this effect size with 95% power when using a repeated measures ANOVA (within-between interaction) and a significance level (α) of 0.05. In correspondence with the stakeholders involved in this research project we accounted for an expected 30% dropout rate including both study dropout and natural dropout due to school change or migration. Therefore, the sample size per school is estimated at 73 children per school. Based on this power calculation we assume that the initially targeted population at baseline (*N* = 477) at the four schools is sufficient to detect a relevant change on the primary outcome measure.

#### Data analysis

To investigate whether the Stadsscholen020 program leads to significant changes on primary and secondary outcomes over time, repeated measures ANOVAs and linear mixed models analysis techniques will be performed for this the longitudinal data. The linear mixed models technique is able the handle missing data using a likelihood based approach, every observation for every participant is used, irrespective of their missing data. The appropriate covariance structure, which accounts for the within-subject correlation, will be chosen based on the model fit using the − 2 log likelihood value. Cross-sectional data will be analysed by comparing means between the different levels (school, neighbourhood, district, and city level) using independent *t*-tests and ANOVA.

## Discussion

To promote equal chances of children growing up in deprived neighborhoods and to decrease long-term consequences of poverty the municipality of Amsterdam initiated a school-based interdisciplinary intervention (Stadsscholen020). The current study aims to evaluate this intervention program at four primary schools in a deprived neighborhood. The implementation of a school-based interdisciplinary approach via primary schools combining the cross-sectoral domains health, poverty, and academic achievement is innovative and the knowledge about the effectiveness is currently limited. Research on this complex intervention approach contributes to more insights on effective ways to decrease inequalities of children growing up in poverty. Using a mixed-methods design among three study parts we intent to examine the potential benefits of the intervention on a variety of outcome measures. Conducting a study in a multi-ethnic sample is a strength of our study and an important step forward in intervention research since these populations are difficult to include as participants in research studies. Another strength of the current study is including a process evaluation. Since, the evaluation focuses on a complex intervention and the current study lacks randomization of the participating schools, causal relationships between interventions and outcomes are difficult to determine. The process evaluation will contribute to these methodological limitation in order to interpreted our findings on the outcome measures and will provide more detailed knowledge about the implementation processes.

However, this study has a number of other limitations which may threaten to value and validity of our study. First, a potential limitation of our study is the large variety of activities and interventions that take place at the schools during the Stadsscholen020 intervention period. It will be a challenge to describe all these activities and interventions into sufficient detail and to monitor the implementation of them separately. Because an interdisciplinary approach can be hampered by the absence of an interdisciplinary or inter-sectoral focus and lack of communication between parties. Therefore, the process evaluation and a close collaboration with the practice and policy stakeholders is important to help us addressing these concerns and to identify strengths and barriers in the implementation process. In order to identify successful components of the interdisciplinary approach, we will describe what is happening at the schools as detailed as possible. Second, since the ethnic composition of the population in the neighborhood is very specific, this can affect the generalizability of our findings to other populations and neighborhoods. Third, a limitation often present in research on complex and interdisciplinary interventions is that the time period of our research project may be too short. Yet, we do not know how long it takes to observe significant changes on the child outcomes. Therefore, it is possible that Stadscholen020 leads to changes on the longer term, that are not already manifested during the time frame of our research project. Despite these limitations, study outcomes are informative to both policy and practice aimed at minimizing unequal chances of children growing up in deprived neighborhoods and help them to start a successful academic career early in their lives.

## Abbreviations

CITO: Centrale Eindtoets Basisonderwijs (Final Examination for Primary Education)
DUO: Dienst Uitvoering Onderwijs (The Education Executive Agency)
FAS: Family Affluence Scale
GGD: Public Health Service of Amsterdam
OIS: Department of Research, Information, and Statistics of the municipality of Amsterdam
OKT: ouder-kind team (parent-child team)
SDQ: Strengths and Difficulties Questionnaire
TSES: Teacher Sense of Efficacy Scale
